# Experience of psychologists in the delivery of cognitive behaviour therapy in a non-western culture for treatment of substance abuse: a qualitative study

**DOI:** 10.1186/s13033-022-00566-3

**Published:** 2022-11-28

**Authors:** Abrar Hussain Azad, Shahzad Ali Khan, Ijaz Ali, Hina Shafi, Nisar Ahmed Khan, Shaaray Abrar Umar

**Affiliations:** 1 Health Services Academy, Islamabad, Pakistan; 2 Community Medicine, Mohi-Ud-Din Islamic Medical College, Mirpur, Azad Kashmir Pakistan; 3 Community Medicine, Abbottabad International Medical College, Abbottabad, Pakistan; 4 Rawal Institute of Health Sciences Islamabad, Islamabad, Pakistan

## Abstract

**Background:**

Psychotherapy is the preferred form of treatment for psychological disorders worldwide. Cognitive behaviour therapy (CBT) is one of the most widely used psychotherapies due to its proven efficacy for psychological disorders, including substance abuse. However, CBT was developed in the West according to the culture of developed countries. Therefore, it requires cross-cultural adaptation for non-Western countries. Pakistan is one of the developing non-Western countries where substance use disorders are increasing at an alarming rate. Despite the proven efficacy of CBT for substance use disorders, there is a dearth of its utilization in Pakistan. Therefore, in the present study, in-depth qualitative interviews were conducted with CBT practitioners in Pakistan to understand barriers and challenges in this regard. The study was a part of a broader project aimed at cultural adaptation of CBT for people with substance use disorders (SUDs) in Pakistan.

**Methods:**

In-depth qualitative interviews were conducted with CBT practitioners (N = 8) working in rehabilitation centres and hospitals in Islamabad, Pakistan. Thematic content analysis was conducted to develop core themes from the data.

**Results:**

CBT for SUDs requires some adjustments according to Pakistani culture for successful utilization. The challenges in providing CBT for SUDs revolved around three main themes, i.e., the mental health system, societal practices, and therapeutic issues, and 10 subthemes.

**Conclusion:**

In order to utilize the benefits of CBT for SUDs in Pakistan, cultural adaptation is necessary as an initial step. However, its delivery requires stringent modifications in the health care system to address these challenges.

## Background

Cognitive behaviour therapy (CBT) has been reported to be one of the most effective psychological interventions for various psychological disorders, including anxiety disorders, depression, obsessive–compulsive disorder, bipolar disorder, and substance use disorders (SUDs) [1–7]. The basic assumption of CBT is that individuals’ thought patterns and beliefs influence their feelings and behaviours [8, 9].

CBT as an evidence-based therapy for the treatment of SUDs has proven efficacy [10]. In particular, it focuses on cognitive and behavioural distortions. However, cognitive distortions are the hallmark of psychological disorders, as there lies a discrepancy between perceptions of information, its processing and reality [1]. CBT can be used both in group and individualized formats, but it has been reported to be more effective in individualized sessions [11]. The perception of psychological disorders and approaches to seeking counselling or psychotherapy for treatment may vary culturally. However, there is a dearth of research exploring such differences. Likewise, insufficient evidence is available regarding use of CBT in developing and underdeveloped countries [12]. Such data could guide CBT practitioners towards adapting CBT for use in their own culture. Lack of research in this regard necessitates exploration and documentation of cultural adaptations and modifications of CBT. It ultimately enables the formulation of a standardized, culturally adapted version of CBT to enhance benefits.

Pakistan is a non-Western developing country with a high prevalence of SUDs. The United Nations Office on Drugs and Crime [13] highlighted in its survey report that 6.7% of individuals used an illegal drug in the preceding year, while 4.3% suffered from SUDs and required urgent treatment, with rates accelerating. For example, according to estimates by the United Nations Office on Drugs and Crime (UNODC) and the Ministry of Interior and Narcotics Control Division Government of Pakistan, in 1980, the use of some sort of substance was prevalent among 50,000 individuals, which rapidly increased to 6.2 million in 2006 and 8.1 million in 2011 [13]. The use of heroin increased markedly in Pakistan over a decade, i.e., from 1980 to 1990 [14]. According to a joint report of the Ministry of Narcotics Control Pakistan and the United Nations Office on Drugs and Crime [13] on drug use in Pakistan, cannabis was the most commonly used drug among individuals aged between 15 and 64 in Pakistan. At the same time, one percent of the population widely used opiates (i.e., opium or heroin) [15].

In 2011, 4.25 million people with SUDs in Pakistan required interventions and treatment; however, only 11.2% of them sought it [16]. This highlights the high disease burden and low rate of treatment among this population. In Pakistan, substance abuse is common among people with lower educational and socioeconomic backgrounds, starting at the age ≤ 18 years [17]. It is taboo in the young age group [18], and women are stigmatized even more [19]. Curiosity and recreation are the major causes of substance misuse, followed by life-changing events and peer pressure [17]. Most people with SUDs seeking treatment are chronic drug users [20]. However, relapse is very common among this population. A major factor for relapse is reported to be contact with peers with substance misuse. In addition, the nonavailability of counselling during treatment is regarded as one of the causes of relapse [17]. Therefore, it signifies the role of counselling in the sustainable treatment of SUDs.

No authority exists at the national level for the registration of psychologists. The Pakistan Psychological Society and Pakistan Society of Clinical Psychologists are two independent organizations that register psychologists. Psychologists work mostly in private organizations, including rehabilitation centres, and some psychologists work as medical social workers in community medicine departments of medical colleges. Government-run hospitals have announced a few posts for psychologists in their psychiatry departments, while there are no psychology departments in hospitals. Rather, there is typically one psychologist’s post in the psychiatry department of each hospital. Psychologists are part of psychiatry departments and work under the supervision of psychiatrists. Sometimes psychologists are involved in history taking and preliminary assessment procedures in the hospital’s outpatient department. The Pakistan Medical Council, the regulatory body of doctors, has recently emphasized establishing psychology departments in teaching hospitals.

SUDs are psychological disorders [21] that require psychological treatment. However, despite the increase in SUDs at an alarming rate in Pakistan, there is a dearth of studies on the utilization of CBT in this regard. Although practising psychotherapists adapt psychotherapies for psychological disorders according to the cultural and religious requirements of society in Pakistan, there is a lack of documentation of such attempts. Major challenges faced by psychotherapists in Pakistan include the stigma attached to mental illness, seeking treatment, and the joint family system. Due to the stigma associated with mental illness, family members consider that consulting a psychotherapist will negatively impact the family’s image in society. Female patients are affected more by such an approach than males [22].

**Fig. 1:**
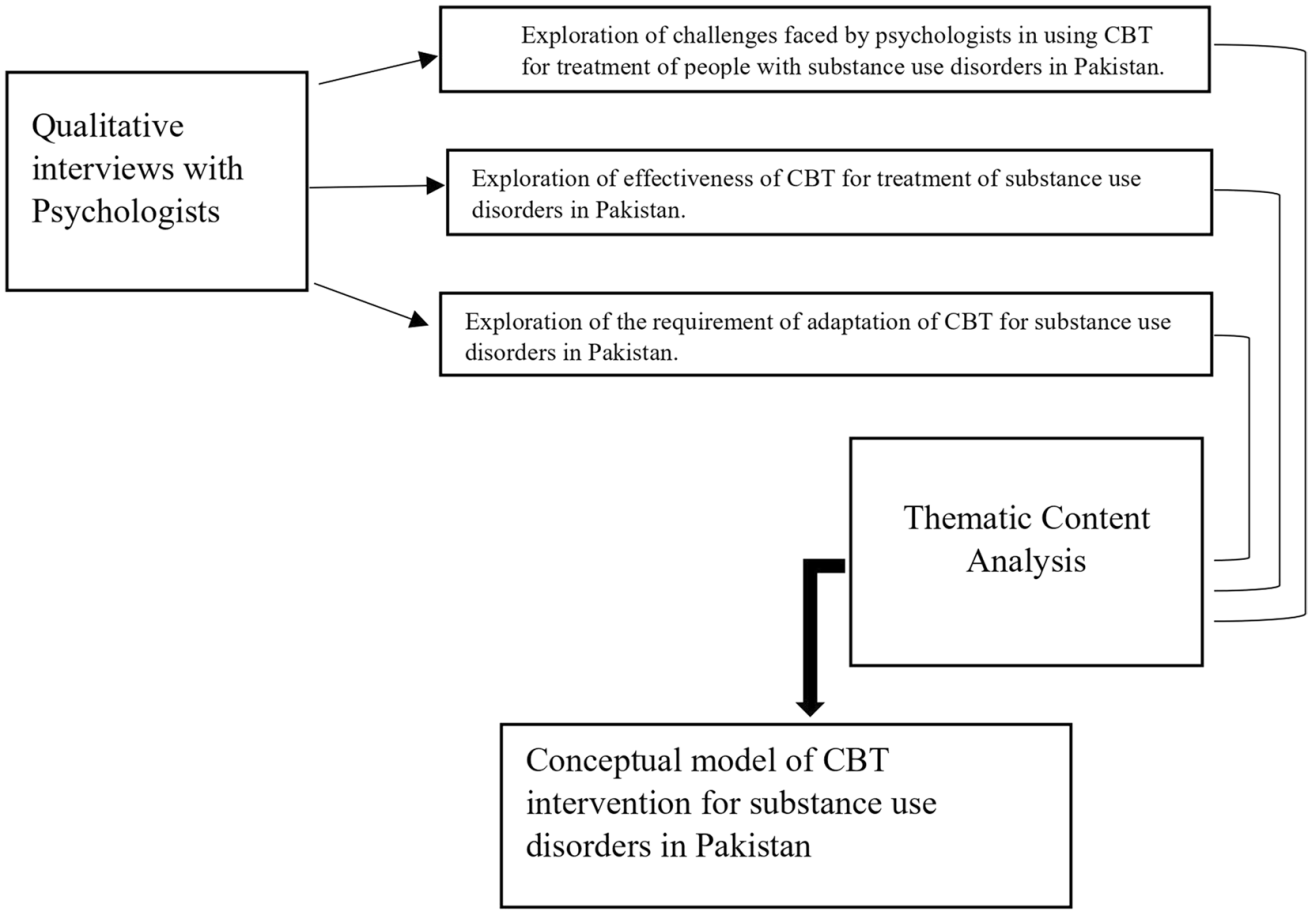
Conceptual framework to assess the utility of CBT for substance use disorders in Pakistan

The current study was conducted as part of a series of studies to discover facts that can help culturally adapt CBT for SUDs in Pakistan. CBT is culturally sensitive [12], therefore, there is requirement to know the cultural factors to adapt it for substance abuse in Pakistani. Ethical approval was obtained from the institutional committee. Thematic content analyses of qualitative interviews was conducted with local psychologists. The research has resulted in points to be considered for adapting CBT for SUDs in Pakistan. The study sought to explore whether CBT is suitable for treating SUDs in Pakistan and presents qualitative findings about the experience of CBT practitioners in using this therapy for persons with SUDs. More specifically, the paper explores the challenges in delivering this therapy and makes suggestions for modification and adaptation of the CBT technique as a treatment option for persons with SUDs in Pakistan. The following conceptual framework was developed to achieve the objectives of the present study (see Fig. 1).

## Methods

### Sample

The study included a sample of clinical psychologists practising CBT (N = 8) for SUDs in Islamabad, Pakistan. The sample was recruited from rehabilitation centres for substance abuse and hospitals. It included male and female CBT practitioners; however, it was predominantly female-oriented (N = 7), as there is a greater number of female psychologists in Pakistan than males. The inclusion criteria was: a postgraduate qualification in psychology, experience of CBT practice for no less than two years, and the experience of CBT delivery to people with SUDs for no less than one year. Participants were approached through rehabilitation centres, hospitals, and through local and social media publicity. Initially, 13 clinical psychologists volunteered to participate in the study; however, only 10 were shortlisted and met all the inclusion criteria. Among them, only eight were interviewed, as a saturation point was achieved. Information pertaining to age, sex, highest qualification, years of CBT practice, and years of working experience with people with substance abuse was collected from the participants.

### Instruments

The study included an interview guide for qualitative interviews.

#### Interview guide

An interview guide was developed to derive maximum information from CBT therapists based on their experience of using CBT to treat SUDs. Questions were designed to attain information regarding barriers and facilitators in using CBT with SUDs. It also explored the modifications or adaptations they made in CBT techniques relevant to Pakistani culture. The interview guide included six open-ended questions involving patients’ problems, psychologists’ cultural and logistic limitations in the delivery of CBT to people with SUDs, factors that facilitate therapy, and modifications/adaptations, if any, in CBT techniques in the context of Pakistani culture and their impact on the outcome of therapy.

### Data collection

Before conducting qualitative interviews to collect data, informed consent was obtained from all participants. All interviews were audio recorded, which participants consented to. Participants were requested to provide as much information as possible on each issue being asked about. The researcher carried out all eight interviews, which were conducted at the psychologists’ workplace at their convenience. The time for data collection was coordinated and agreed upon ahead of the interviews. The respondents were free to impart as much information as they preferred. Probes and follow-up questions were also asked in the interviews to ensure the flow of the discussion. The respondents occasionally overlapped information while responding to different questions. It was ensured that all questions and topics listed in the interview guide were addressed and not missed during the interview. Each interview was completed in 45–60 min on average.

### Data analyses

Data were analysed using the six-step framework of thematic content analysis [23]. Data analysis was driven by a combination of both thematic (top-down) and inductive (bottom-up) approaches [23], as it included data regarding specific questions and additional information that CBT practitioners provided.

All the interviews were transcribed verbatim in the *first step*. In the *second step,* each interview was coded and data which fitted together was organized into meaningful sub-themes resulting in 35 subthemes. In the *third step,* interrelated subthemes were divided into five broad themes, i.e., flaws in the health system, legal issues and social insecurities, patients’ perspectives, challenges for psychologists in the delivery of CBT, and religious practices and familial norms.

In *step four,* it was ascertained that the themes and subthemes were interrelated and accurately represented the data. After detailed discussions and in-depth analysis, data was kept under the subthemes that appeared to be most closely associated with it, resulting in 10 subthemes as some became redundant and eliminated. In addition, two themes, religious practices and familial norms, and legal issues and insecurities, were not found distinct enough to represent two different themes. Therefore, they were subsumed and renamed as cultural and social issues resulting into four main themes, i.e., flaws of the health system, cultural and social issues, patients’ perspectives, and challenges for psychologists in the delivery of CBT, and 10 sub-themes.

**Fig. 2:**
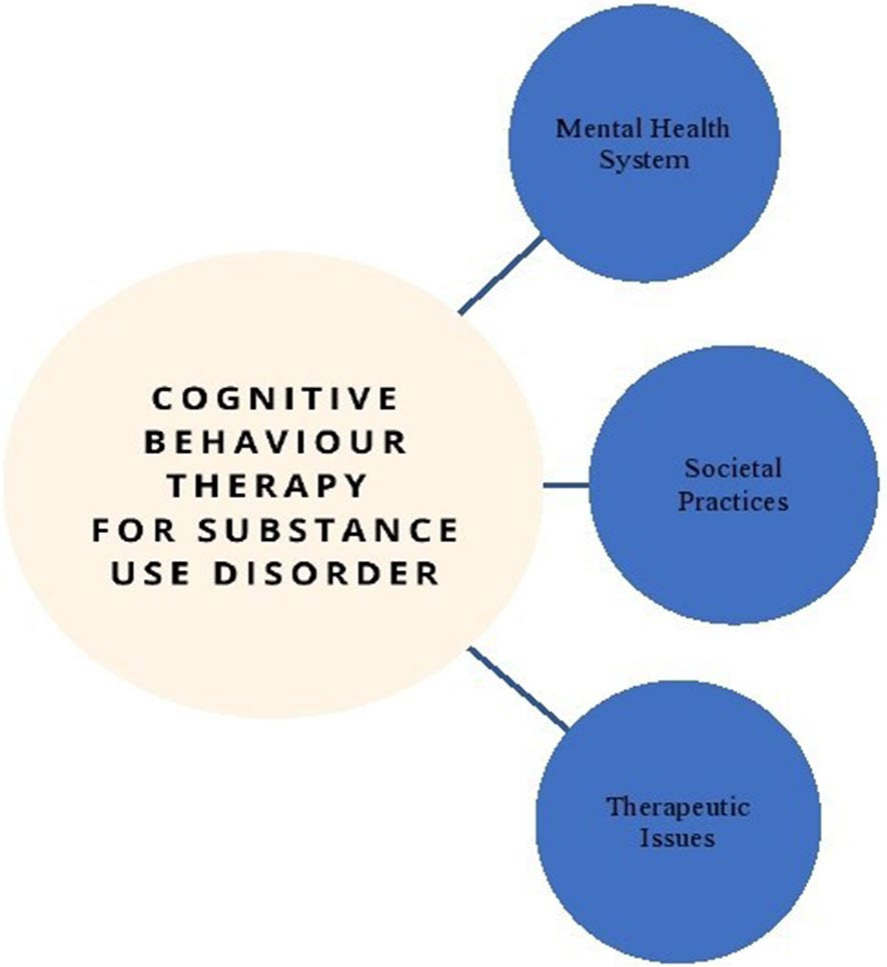
Thematic map

In *step five,* two themes, flaws of the health system, and cultural and social issues were renamed, while two themes, patients’ perspectives, and challenges for psychologists in the delivery of CBT were subsumed. It resulted into three main themes i.e., mental health system, societal practices, therapeutic issues and 10 subthemes. See Fig. 2 for the thematic map of the study and Table 1 for an overview of themes and subthemes derived from the present study. Finally, *step six,* i.e., data reporting is illustrated in the results section below.

**Table 1:** Themes and categories derived from the study

Main themes	Categories
Mental health system	1. Logistic issues 2. Collaboration between psychologists and psychiatrists
Societal practices	1. Belief system 2. Influence of Family and Peers 3. Illegal practices and drug mafia
Therapeutic issues	1. Perceptions of patients’ understanding of CBT 2. Issues relating to patient engagement with CBT and attrition rates 3. Perceptions of patients’ compliance with CBT therapeutic processes 4. Psychologists’ perceptions of personal challenges 5. Useful CBT techniques

## Results

Eight CBT practitioners (i.e., seven females and one male) were recruited for the present study. Their ages ranged from 27 to 43 years. Among them, one had a PhD in psychology, three had MS in psychology, and five had MSc and a postgraduate diploma in clinical psychology. Years of practising CBT ranged from three to 11 years, while the experience of working with people with SUDs ranged from four to seven years. Although, the data were collected from CBT practitioners/psychologists, they provided ample information regarding various aspects of society and systems affecting the delivery of CBT as a treatment for SUDs. See Table 1 for themes and subthemes that were identified during the analyses. However, its detailed description is illustrated below.

### Mental health system

The mental health system was divided into the following subthemes:

#### Logistic issues

Enormous discrepancy between workforce i.e., CBT practitioners and workload was reported. *“On a daily basis, I see 20 patients on average. In addition, the workload is not even uniform. Some parts of the day, I see 4–5 patients in an hour and some parts of the day I only see one patient per hour, it depends on the arrival of the patients. It definitely affects my work badly. Sometimes I am not at all happy about it, as I am not able to utilize my ability fully.”* (Female, 6 years using CBT).

It was highlighted that a greater workload leaves CBT practitioners with limited case preparation time, resulting in frustration. *“It becomes very frustrating when you want to contribute to the mental health of people, but limited time does not allow you to.”* (Female, 6 years using CBT). It was also reported that shared working space for CBT practitioners affects the privacy of the patients, which is a basic requirement for psychotherapy. *“Patients with psychological illnesses always require confidentiality and privacy to share their personal issues, because these are their sensitivities and vulnerabilities and they don’t want to open up in front of everyone. It is unlike any general medical condition, but it is unfortunate that psychologists not only have to share their working space with their colleagues, but there are large number of patients also queuing for their turn, which makes it very difficult for our clients to entrust us with their problems.”* (Male, 5 years treating SUDs).

It was also highlighted that access to treatment was challenging for people living far away from Islamabad, where better treatment facilities were available. *“Family members sometimes bring their relative with substance issues to the hospital a day ahead of the appointment. They travel from various distant places, and it is not possible for them to be on time during the working hours. Therefore, they have to come a day ahead of the appointment. Then, they stay on the premises of the hospital the night before their appointment, as they cannot afford any private accommodation nearby for a night’s stay. Such odds in themselves are traumatizing for the whole family. Such issues make it hard for the majority to avail treatment for people with substance abuse.”* (Female, 10 years using CBT).

#### Collaboration between psychologists and psychiatrists

Power imbalance between psychiatrists and psychologists at workplace and a lack of understanding of psychotherapy on part of psychiatrists was reported. *“At times it becomes difficult to make CBT understandable not only for the patients, but also for your psychiatry colleagues.”* (Female, 4 years using CBT). *“I assist senior psychiatrists in the hospital. I am sometimes answerable to my boss for spending more time with certain patients, which affects my decision-making and independence to formulate therapy according to the requirements of the patients.”* (Female, 3 years using CBT).

### Societal practices

Some societal practices were highlighted that restrict the utilization of CBT for SUDs in Pakistan. The following subthemes were identified:

#### Belief system

The interviews highlighted the effects of a deep-rooted belief system on the treatment approach towards SUDs. *“I have seen patients with chronic substance abuse with family members stressing the point that their relative has been taken to various renowned spiritual healers and “Dargahs” (shrines), but to no avail.”* (Male, 7 years using CBT). A family member’s saying was quoted, *“he prefers staying in the nearby “Mazaar” (shrine). He doesn’t eat the meals we give him, and he doesn’t return home for days despite our requests. People who visit “Mazaar” ask him and others staying there to pray for them.”* (Female, 5 years using CBT).

#### Influence of family and peers

It was reflected in the interviews that family support was essentially required for the success of therapy. However, the family can have a negative effect on therapy outcomes. *“A lack of tough love on part of family members is what ruins all the efforts put into therapy for people with substance abuse. Family simply gives in to the begging of their relative and for their “release” from treatment.”* (Female, 7 years treating SUDs). *“There are many instances when I requested family for assisting patients in their homework, motivating them to change, or having a diary of automatic thoughts, cues and triggers for using drugs. However, family members appeared to be more controlling than supportive, which offended and discouraged the patients from becoming self-aware and self-reliant.”* (Female, 5 years using CBT).

The prevalence, process, and causes of substance abuse among the youth were reported by the respondents. *“Youngsters normally start taking drugs for fun or for being accepted among their peers. Initially, it is kept secret from family or parents, as such behaviour is completely unacceptable in society. That is the reason the issue cannot be addressed in a timely manner. It [the addiction] gets full bloom before being discovered by the family.”* (Male, 5 years treating SUDs). *“It is correct that causes of drug abuse vary from person to person, but it’s my observation that adolescents with low self-esteem are more susceptible to peer pressure and indulging in problematic behaviours, such as substance abuse or drug addiction. Therefore, work on their self-esteem is an initial step in the therapy.”* (Female, 7 years treating SUDs). Almost all the participants agreed that educated youth with substance abuse who became addicted due to peer pressure were easy to engage in CBT. Therapy outcomes were also positive with such individuals.

#### Illegal practices and the drug mafia

Certain illegal practices associated with drug misuse that hinder the treatment process for people with SUDs were reported: *“it is not always the impulse or withdrawal effect for the patients which makes them relapse, but they are so deeply ensnared in drug mafias, including legal personnel or drug dealers, that they cannot be helped despite their own and their families’ desires.”* (Male, 5 years treating SUDs). *“I can feel how scared they [patients] feel, through their nonverbal cues when asked to talk about the high-risk situations for drug abuse. Most likely, it is the flawed law enforcement system that victimizes them instead of helping them.”* (Female, 4 years treating SUDs).

### Therapeutic issues

Therapeutic issues involved the following subthemes:

#### Perceptions of the patients’ understanding of CBT

It was reported in the interviews that reliance on psychotherapy seemed impractical, unrealistic or illogical to the patients. *“The patient asked me with surprise and a little bit of annoyance, do you think my problem is so light to be treated with talk only?”* (Female, 4 years using CBT). Moreover, it was highlighted that lack of awareness about usefulness of psychotherapy hinders its acceptability. *“People with substance abuse are full of uncertainties regarding CBT as a treatment option because it is a completely new idea for them. They do not seem to understand how talking about the problem or following some tasks or exercises can be helpful in treating their issues. They do participate in therapy half willingly, get bored with it very soon, and terminate therapeutic treatment shortly after.”* (Female, 3 years using CBT).

The patients’ lack of understanding of the basic concepts of CBT was also reported. *“One problem which I encounter most of the time is that the patients argue that their beliefs are based on their religion, which is perfect and without any distortions or flaws. Explaining it to them that the beliefs I am talking about are not religious, rather individual and personal, is time-consuming and tedious task.”* (Female, 9 years of using CBT).

Likewise, difficulties in understanding the concepts of delaying, distracting, decatastrophising, and disputing were reported: *“(patients) consider that delaying means delaying the urge to use the drug for some time as a formal requirement of therapy, after doing so they feel liberated from the task assigned to them and start thinking about the drug all over again, which is surely counterproductive.”* (Female, 5 years using CBT). *“Distractions are often misperceived. People with substance abuse try to distract themselves from the urge to use the drug with something or someone related to drug, which obviously is not helpful.”* (Female, 8 years using CBT). *“Sometimes there is lack of self-awareness among people with substance abuse. They believe that they are using the drug only for recreational purposes, and they do not consider it catastrophic if they resist the urge to use the drug or if the drug is not available. However, guided discovery often reveals the contributing role of the catastrophizing aspect of their thinking.”* (Female, 7 years treating SUDs). *“Disputing may be due to its negative connotation taken as something undesirable. I have had patients ask me if it is required to be aggressive towards the urge or thought to use the drug because it causes them more anxiety and guilt when they do so.”* (Female, 11 years using CBT). In addition, it was reported that it was equally difficult to make the concept of irrational beliefs understandable to the patients. A patient’s response was quoted: *“… why would I have an irrational belief? I am educated, and I know my problems are caused by my life circumstances and not by any external force.”* (Female, 7 years using CBT).

#### Issues relating to patient engagement with CBT and attrition rates

Engaging patients in therapeutic sessions was reported to be challenging. *“There are more male patients with substance abuse compared to females, while most CBT practitioners or psychologists are females. Perhaps the male patients are not comfortable engaging in a long therapy session with a female practitioner due to cultural norms or hesitation. That’s why they try to end the session quickly. This has no positive effect on their treatment though.”* (Female, 7 years treating SUDs). *“People coming to hospitals in Islamabad for treatment normally travel long distances in the public transport, which involves a lot of time and effort. This probably causes a rush, as they have to travel back a long distance again. They think therapy should be like getting a doctor’s prescription quick and crisp, which is not the case.”* (Female, 5 years treating SUDs).

“*Only those patients who are very desperate to share the underlying causes of their addictive problems have higher acceptability for CBT. It provides them the opportunity to be heard and share, which is an initial step towards success of the therapy.”* (Female, 6 years using CBT).

*“Females are more willing to accept CBT as a treatment option compared to male patients.”* (Female, 4 years using CBT).

Limited capacity for payment, and uncertainties regarding CBT as a treatment option, also affected patients’ engagement in CBT. *“If a patient has to pay 100 rupees for a session and the same amount for the doctor’s appointment, he’ll prefer the doctor’s appointment and refuse to participate in the therapy.”* (Female, 5 years using CBT). *“One of the patients argued I’d prefer taking some edibles home with the money instead of giving it away for mere talk.”*(Female, 5 years using CBT).

The attrition rate was reported to be lower among people with substance abuse who were residing in the rehabilitation centres compared to those who visited the outpatient department in the hospitals: *“most of the substance abuse clients discontinue therapy after two or three sessions, taking it nowhere,”* (Female, 7 years treating SUDs).

*“Patients in rehabilitation centres have to follow a certain routine, and they take therapeutic sessions as part of their routine activities, so it is easy to engage them in sessions of CBT during their stay in the centre.”* (Female, 4 years treating SUDs).

*“Commitment to therapy is a heavy responsibility requiring persistence, but there is a great risk of relapse in substance abuse of clients. It makes it hard for them to continue their therapy.”*(Female, 6 years treating SUDs).

*“Patients think psychotherapy is some sort of magic which will eradicate substance from their lives without any of their input or effort.”* (Female, 4 years treating SUDs). *“Patients often have a long history of substance abuse but they want a quick fix. Such unrealistic expectations can take them anywhere from disappointment to discontinuation of therapy.”* (Female, 7 years treating SUDs).

#### Perceptions of patients’ compliance with CBT therapeutic processes

Compliance with CBT homework tasks was reported to be low in the present study. *“I can see from the face of the patient that they are not going to do their homework as they seem not to be interested in taking it. Therefore, I repeatedly explain to them how important it is for them to cooperate if they truly want to improve.”* (Female, 5 years using CBT). *“When I feel that the patient is not going to follow what they have been asked for as homework, I involve their family members to facilitate completing their homework.”* (Female, 4 years using CBT).

*“Patients sometimes expect an immediate remedy for the deep-rooted and prolonged disorder, which is why they find it hard to work on a weekly basis to achieve relief. Maybe it is the despair, impulsivity, or finding it absurd to do homework, which is at work when not complying with the suggestions.”* (Female, 11 years using CBT).

Moreover, in the present study, CBT practitioners highlighted some confusion, which patients experienced during the therapy, which if not addressed appropriately, resulted in dropouts or relapse. *“I have found patients worrying and guilty about quitting the drug not because it was hard for them to quit, but they were, in fact, equating it with leaving their friends and company in which they used to do drugs. In such cases, proper guidance is necessary for differentiating between quitting the drugs and leaving the friends or the company of peers.”* (Female, 4 years treating SUDs). *“It is not unusual for people with substance abuse to have occasional lapses during therapy, but sometimes patients consider it their failure and become discouraged and demotivated. Such demotivation, if not resolved in a timely manner, can result in a complete relapse. We encourage patients to view lapses as opportunities to identify the weaknesses, cues, and triggers, which led to a lapse to avoid relapse.”*(Female, 6 years treating SUDs).

In addition, patients’ conformity bias and misreporting were reported to be counter effective for CBT: *“at the start of the therapy patients tend to be very cooperative and motivated, maybe they are trying to be accepted and to be more presentable. They are keeping their masks tight. They probably try to guess what will be appreciated in the therapy so they fake report the positives and negatives about substance abuse. Such overreporting affects the whole process and progress of therapy negatively.”* (Female, 5 years using CBT).

Likewise, a poorly reported history of drug abuse and relapses was reported to be another factor that negatively affected the outcome of the therapy: *“knowledge of the history of substance abuse, number of times the patient sought the treatment or psychotherapy, and instances of relapses in the past are crucial for the psychotherapist to determine the goals of therapy and the selection of appropriate therapeutic techniques for the patient, but patients do not realize how important it is for their treatment. Their distorted reporting hinders the process of abstention.”* (Female, 6 years treating SUDs).

Similarly, it was reported that some facts patients did not report correctly may facilitate the therapeutic process:. *“patients have a hard time exploring the internal factors responsible for their substance abuse behaviour. As a therapist, we can only guide them through such introspection, although it depends completely on their own sincere efforts to explore.”* (Female, 7 years treating SUDs).

#### Psychologists’ perceptions of personal challenges

Furthermore, practical issues constraining the use of CBT were also highlighted in the interviews: *“standardized CBT manuals are available for different types of substance abuse, but there is no standard translation available in Urdu [Pakistan’s national language] for any one of them. This limitation restricts us [psychotherapists] from implementing the techniques of CBT and accomplishment of homework forms.”* (Female, 6 years treating SUDs). *“It becomes a great challenge to translate all assessment tools and worksheets for homework for patients. We [psychotherapists] have a short amount of time due to massive workload and limited therapists available.”* (Female, 3 years using CBT).

Making CBT terms understandable for patients was reported to be challenging as well: *“when I tell my clients that you have to work on your absolutistic thought patterns. You have to start changing “should” and “musts” patterns in your thinking. My patients, most of the time, argue that they are very compromising, and they never insist on having anything. They do not understand that it’s not about having material gains, but it is about changing the attribution processes.”* (Female, 4 years using CBT).

In addition to challenges pertaining to patients and the system, certain limitations on the part of the therapists were reported to have a negative effect on the therapeutic process and outcomes: *“I understand that empathy is vital in the treatment of people with substance abuse. I also admit that when I am overburdened, as I am the only psychotherapist working in my organization, it becomes extremely difficult for me to empathically involve my patients, which definitely affects therapy outcomes negatively.”* (Female, 3 years using CBT).

Additionally: *“due to time constraints and being overworked, it is hard to keep updated the therapeutic knowledge. I feel bound to use traditional CBT techniques.”* (Female, 5 years using CBT). Furthermore: *“CBT takes a collaborative approach, while patients expect directions or advice from us (therapists). They request it until they understand that therapy has to be collaborative. I do suggest and direct them on certain issues in order to move on in therapy.”* (Female, 4 years using CBT).

#### Useful CBT techniques

In the context of Pakistan, certain CBT techniques were reported to be more effective: *“providing CBT in group format has resolved many issues for me, like time constraints, patients’ discomfort and inhibition to share their problems, rapport building, and ambivalence to change or lack of motivation to start therapy. Group format probably gives them confidence that they are not alone in their situation.”* (Female, 11 years using CBT).

*“I spend a lot of time motivating those patients who are brought to therapy by their family or referred by law enforcement agencies. Motivation is key to change; once patients are motivated to change, it becomes easy to proceed with therapy and succeed.”* (Female, 8 years using CBT).

*“I found role play as one of the most effective therapeutic techniques. It is equally useful for patients with a low educational background. This is similar to real life situations. The participants were usually people with substance abuse under therapy. It facilitates learning life skills of assertiveness, resistance, and confidence simultaneously for all the participants, hence is very efficient.”* (Female, 5 years using CBT).

*“Successfully educating patients about their automatic thoughts, irrational beliefs, misconceptions about therapy, exploration of triggers and cues for drug usage, and possibility of quitting the drug by changing their thought patterns helps a lot in making therapy a success.”* (Female, 7 years treating SUDs). *“Training and practice of progressive muscular relaxation is very useful in distracting and relaxing patients during cravings for drugs. It takes time to train patients but once learned it has greater benefits.”* (Female, 4 years treating SUDs).

*“I do some modifications in the homework worksheets and forms on my own to make them understandable for patients who cannot read or understand it in English. I make sheets more illustrative and interactive by adding pictures and images to make the homework understandable.”* (Female, 4 years using CBT).

## Discussion

The aim of the present study was to explore the experiences of psychologists in the delivery of CBT for the treatment of SUDs in a non-Western context. Three main themes, mental health system, societal practices, and therapeutic issues, along with 10 subthemes, were identified. The subthemes included logistic issues, collaboration between psychologists and psychiatrists, belief systems, influence of family and peers, illegal practices and the drug mafia, perceptions of patients’ understanding of CBT, issues relating to patient engagement with CBT and attrition rates, perception of patients’ compliance with CBT therapeutic processes, psychologists’ perception of personal challenges, and useful CBT techniques.

In the themes and subthemes, a number of barriers and facilitators to the use of CBT in Pakistan were identified. Family of people with substance abuse was found to be one of the major facilitators in the delivery and success of CBT. For example, families who supported their relative to stay in rehabilitation and complete the medical and psychological treatment enabled the person with substance abuse to recover. Families have been reported to bring patients to therapy sessions, support homework completion, and assist in keeping records of cues and triggers for using the substance. Therefore, the delivery of CBT was much easier to complete and attain its goals for people with substance abuse who had cooperative understanding and supportive family members. The positive contribution of family support to the success of therapy has also been reported earlier [24]. Similarly, a strong parent-adolescent relationship has been shown to reduce substance use in adolescents [24]. In contrast, lack of family support resulted in higher relapse among people with SUDs [25]. However, it is also vital that families must be educated about the difference between support and control, as control could be counterproductive. Their awareness may be enhanced by educating them at the start of therapy. The requirement and positive effects of educating parents to support the treatment of SUDs among their children have been established [26].

In the present study, it was found that educated people with substance abuse, such as students from colleges and universities, had a higher positive response towards and acceptance of CBT than those with no or less education. Although the level of education cannot be increased, attempts may be made at the start of the therapy to at least educate the person with substance abuse about the negative effects of substance abuse and effectiveness of CBT to counter them. An earlier qualitative study [27] showed that young adults who were not educated about the potential adverse effects of addictive substances were more inclined to abuse substances. Educational reforms were suggested in this study [27] for the awareness of youth about the risks of substance use for physical health and psychological well-being.

It was also found that people prefer taking sessions of short duration. Therefore, brief sessions may be planned initially until the patient starts seeing the benefits of the therapy. Afterwards, with the mutual collaboration duration of the sessions may be enhanced. In addition, some of the reported useful techniques were motivation, interactive home-work sheets, role play, relaxation training, and group therapy. These may be utilized to facilitate the delivery and success of CBT for the treatment of people with SUDs.

Patients’ receptivity and acceptance of therapeutic suggestions has been reported to be one of the factors contributing in success of psychotherapy [22]. However, in the present study, one of the barriers to use CBT was found to be more inclination to adhere to the instructions of the medical professional among patients and their family members. This may be because of limited awareness about psychological treatment or more familiarity with medical professionals among patients and their family members. Due to such familiarity, they trust them and consider them credible. Therefore, if encouraged and assured by the physician regarding the effectiveness of therapy, the rate of acceptance of CBT may increase substantially. Positive role of a communicator’s credibility and trustworthiness in attitude change has been supported (Priester & Petty, 2003). This may be utilized to enhance receptivity of CBT among people with SUDs.

In addition to facilitators, a few barriers to the delivery of CBT were identified. The perception of CBT as mere talk is one of the major barriers to acceptance. Therefore, creating more awareness about effectiveness of CBT may increase its acceptance among patients and their families. Moreover, the approach of seeking medical treatment for all illnesses is another barrier, and it may be overcome with increased collaboration between psychiatrists and psychologists. If awareness sessions regarding CBT are provided collaboratively to the person with substance abuse and their family, it may facilitate the delivery and acceptance of CBT.

Superstitious beliefs were also a major reported obstacle that restricted access to psychological treatment. An adverse impact of superstitious beliefs on psychological treatment outcomes in Pakistan was previously reported in two qualitative studies [28]. Superstitious beliefs may be addressed by inviting renowned, admired, and respected religious scholars to advocate seeking psychotherapy for SUDs.

The delivery of CBT in Pakistan is a complex phenomenon that can only be addressed through the collaboration of medical, psychiatric, psychological, and religious compartments of society. Moreover, CBT requires adaptation in terms of standardized translation of its manuals for SUDs in the national language, modifications of homework forms and worksheets, e.g., adding illustrative photos, and implementation of its techniques according to the non-Western culture of Pakistan. It will also enhance the acceptability and understanding of CBT for people with SUDs. Adaptation of CBT may also include clear plans for educating people with SUDs and their families about substance abuse, CBT and its effectiveness for SUDs.

Based on the reported effectiveness of CBT in the treatment of SUDs [1–7] and the factors, facilitators, barriers, and challenges affecting its use in Pakistan, an intervention framework was conceptualized in the present study to enhance its utilization in the Pakistani context. See Table 2 for details.

**Table 2:** Conceptual model for culturally adapted CBT for people with substance use disorders in Pakistan

Conceptual model of CBT intervention for people with substance use disorders in Pakistan	Therapeutic materials	• Translated CBT manuals for substance use disorders in national language Urdu • Translated CBT homework sheets and forms in national language Urdu • Cards, sheets, and charts with illustrative images to make basic therapeutic concepts and patients’ thought processes understandable for them
	Therapeutic processes	• Before formal start of the therapy 1–2 educational sessions should be conducted with patients and their family members to educate them about nature and effectiveness of CBT for substance use disorders. Psychiatrists should also participate in these sessions to increase awareness • Initial few sessions should be short (20–25 min long) until acceptance of long sessions of standard duration, i.e., 40–45 min • Group sessions, and role play may be used in initial sessions to encourage people to open up and develop rapport • Remaining sessions should be of standard duration 40–45 • When collaborative approach does not work it should be alternated with instructional/directional approach
	Therapeutic techniques	• To motivate patients and their families to engage in and comply with the therapy success stories of similar patients should be shared with them during educational sessions before the start of therapy • Progressive Muscular Relaxation techniques should be taught to patients to deal with cravings • Psychoeducation should be continued during the sessions to make therapeutic processes and techniques understandable for patients
	Cultural facilitators	• To counter superstitious beliefs renowned religious scholars’ speeches in favour of medical and psychiatric treatment and negation of superstitious beliefs should be shared with the patient and their families during educational sessions before starting psychotherapy • Families of patients should be engaged in therapy as families play vital role in treatment decisions and processes

The study had a few limitations, such as only including the experiences of CBT practitioners in the delivery of CBT for the treatment of substance abuse. Therefore, all the information regarding patients, their family members, and societal issues came from the therapists. There is a requirement for patients and their family members’ opinions regarding their experiences in availing psychotherapy as a treatment option.

## Conclusion

The study is helpful in policy formulation regarding the treatment of people with substance abuse. It is also useful for future studies to advance treatment options for people with SUDs. It will also enhance knowledge and awareness regarding psychotherapeutic treatment options and their effectiveness for people with SUDs. Such awareness ultimately will be a source of hope for people with SUDs and their family members.

## Data Availability

Data and all the material relevant to the present study is available with the corresponding author, who may be consulted to access it.
